# Lats1 suppresses centrosome overduplication by modulating the stability of Cdc25B

**DOI:** 10.1038/srep16173

**Published:** 2015-11-04

**Authors:** Satomi Mukai, Norikazu Yabuta, Kaori Yoshida, Ayumi Okamoto, Daisaku Miura, Yasuhide Furuta, Takaya Abe, Hiroshi Nojima

**Affiliations:** 1 Department of Molecular Genetics Research Institute for Microbial Diseases, Osaka University, 3-1 Yamadaoka, Suita City, Osaka 565-0871, Japan; 2 Department of Pharmacy, Hyogo University of Health Sciences, Kobe, Japan; 3 Animal Resource Development Unit, 2-2-3 Minatojima-minamimachi, Chuo-ku, Kobe, Hyogo 650-0047, Japan; 4 Genetic Engineering Team, RIKEN Center for Life Science Technologies, 2-2-3 Minatojima-minamimachi, Chuo-ku, Kobe, Hyogo 650-0047, Japan

## Abstract

Numerical aberration of the centrosome results in chromosome missegregation, eventually leading to chromosomal instability, a hallmark of human tumor malignancy. Large tumor suppressors 1 and 2 (Lats1 and Lats2) are central kinases in the Hippo pathway and regulate development and tumorigenesis by coordinating the balance between cell proliferation and apoptosis. Importantly, Lats1 and Lats2 also play pivotal roles in cell cycle checkpoint and mitosis. The Lats proteins localize at centrosomes, but their centrosomal functions remain elusive. Here, we generated *Lats1*-null knockout (*Lats1*^−/−^) mice and established *Lats1*-null mouse embryonic fibroblasts (MEFs). In *Lats1*^−/−^ MEFs, centrosomes were markedly overduplicated, leading to severe mitotic defects such as chromosome missegregation and cytokinesis failure. We also found that Lats1 physically interacts with Cdc25B phosphatase that localizes both at the centrosome and in the nucleus and regulates the linkage between the centrosome cycle and mitotic progression. Although Lats1 did not phosphorylate Cdc25B, loss of Lats1 in MEFs caused abnormal accumulation of Cdc25B protein and hyperactivation of Cdk2 toward nucleophosmin (NPM/B23), one of the licensing factors involved in centriole duplication. Taken together, these data suggest that Lats1 regulates Cdc25B protein level and subsequent Cdk2 activity, thereby suppressing centrosome overduplication during interphase.

The centrosome comprises two centrioles and pericentriolar protein matrix (PCM), which functions as a microtubule organizing center for the formation of bipolar spindles during mitosis and as a basal body for assembly of primary cilia at the quiescent stage during interphase^1^. Structural and numerical aberrations of the centrosome occur in a variety of human tumors^2^,^3^. Supernumerary centrosomes lead to chromosome missegregation, leading to aneuploidy and chromosomal instability, which are hallmarks of human tumor malignancy^4^,^5^. In some diseases including autosomal recessive primary microcephaly (MCPH), ciliopathy, and dwarfism, defective centrosomes with abnormal structure and number have been reported^6^,^7^. Indeed, 10 of 13 MCPH-related proteins (microcephalin, WDR62, CDK5RAP2, ASPM, CPAP, STIL, CEP135, CEP152, Cdk6, and Cep63) localize at the centrosome, and some of them have been implicated in regulation of centrosome number. Therefore, the physiological functions of centrosomal proteins are involved not only in tumorigenesis, but also in diseases accompanied by dysregulation of brain development and control of body size^7^.

Centrosome duplication is regulated by the centrosome cycle, which is divided into four steps: centriole disengagement in early G1 phase, nucleation of daughter centrioles in G1-S phase, elongation in G2 phase, and separation in G2/M phase^1^. The initiation of centrosome duplication is induced coordinately with DNA replication during S phase, which is tightly regulated by the kinase activities of the Cdk2-cyclin E and Cdk2-cyclin A complexes^8^,^9^. The Cdc25 phosphatase family, including Cdc25A, B, and C, is the principal regulator of the activity of the Cdk-cyclin complex during the cell cycle^10^. In particular, Cdc25B localizes to the centrosome throughout the cell cycle^11^,^12^,^13^,^14^ and regulates centrosome duplication during interphase and microtubule assembly during mitosis^11^. Consistent with these functions, overexpression or depletion of Cdc25B causes centriole overduplication or loss of centrosome integrity, respectively, in cultured human cancer cell lines^15^,^16^.

The Hippo signaling pathway, a conserved mediator of growth control and cell fate decision, plays a crucial role in restraining cancer development^17^. Mammalian Large tumor suppressor 1 (Lats1) and Lats2, the main kinase components of the Hippo pathway, phosphorylate and inactivate Yap/Taz, a transcriptional activator of cell proliferation and anti-apoptotic genes. Lats2 localizes to the centrosome/spindle pole and regulates mitotic progression^18^,^19^. Loss of Lats2 in mouse cells causes a wide variety of mitotic errors, including centrosome fragmentation, chromosome misalignment, and cytokinesis defects with multinucleation^20^,^21^. Moreover, centrosome stress induced by treatment of cells with the spindle poison nocodazole causes Lats2 to translocate from the centrosome to the nucleus, thereby preventing polyploidization via the p53 pathway^22^. On the other hand, although human Lats1 also localizes at the centrosomes in human cancer cell lines such as U2-OS osteosarcoma cells and HeLa cervical cancer cells^23^,^24^,^25^, to date no study has described the impact of Lats1 dysregulation on the centrosome cycle. Therefore, the biological role of centrosomal Lats1 and the molecular mechanism by which Lats1 regulates the centrosomal integrity remain unclear.

In this study, we generated *Lats1*-null knockout (*Lats1*^−/−^) mice, and established *Lats1*^−/−^ mouse embryonic fibroblasts (MEFs) from these animals. *Lats1*^−/−^ MEFs exhibited centrosome overduplication. Moreover, loss of Lats1 also led to multipolar spindle formation, chromosomal misalignment, micronuclei, and cytokinesis failure, even when the cells entered M phase. Importantly, Lats1 and Lats2 physically interacted with Cdc25B; however, Cdc25B associated more strongly with Lats1 than Lats2. Notably, Lats1 did not phosphorylate Cdc25B. The interaction between Lats1 and Cdc25B contributed to the destabilization of Cdc25B protein and, subsequently, activated Cdk2, thereby preventing centrosome overduplication. These findings suggest that Lats1 stringently regulates the duplication of the centrosome by restricting irrelevant stabilization of Cdc25B, thereby ensuring that the centrosome duplicates once per cell cycle.

## Results

### Lats1 localizes at the centrosomes during G2/M phase but not during interphase

To elucidate the molecular function of Lats1 at the centrosome, we generated Lats1-null knockout mice by disrupting a part of exon 5 (E5, amino acids 684–853), which encodes the kinase domain (Supplementary Fig. S1A–C online). Both male and female *Lats1*^−/−^ mice exhibited growth retardation (Supplementary Fig. S1D online) and reduced body weight relative to wild-type mice (Supplementary Fig. S1E and F online), suggesting that depletion of Lats1 causes dwarfism in mice; this observation is consistent with the phenotypes of two other types of Lats1-knockout mice generated by truncation at the C-terminus^26^ (Supplementary Fig. S1A online) or N-terminus^27^. However, there was no difference in the incidence rate of spontaneous tumors between *Lats1*^+/+^ and *Lats1*^−/−^ in 2-year-old mice. We established *Lats1*^+/+^ and *Lats1*^−/−^ MEFs from *Lats1*^+/+^ and *Lats1*^−/−^ embryos, respectively. In addition, we performed western blot analysis using two kinds of Lats1 antibodies, CST-C66B5 and Bethyl, which recognize the N-terminal and C-terminal portions of Lats1, respectively; these analyses confirmed that *Lats1*^−/−^ MEFs do not express full-length Lats1 protein or any truncated Lats1 fragments (Supplementary Fig. S1G online). These results demonstrate that the *Lats1*^−/−^ MEFs we generated were Lats1-null cells.

**Figure 1:**
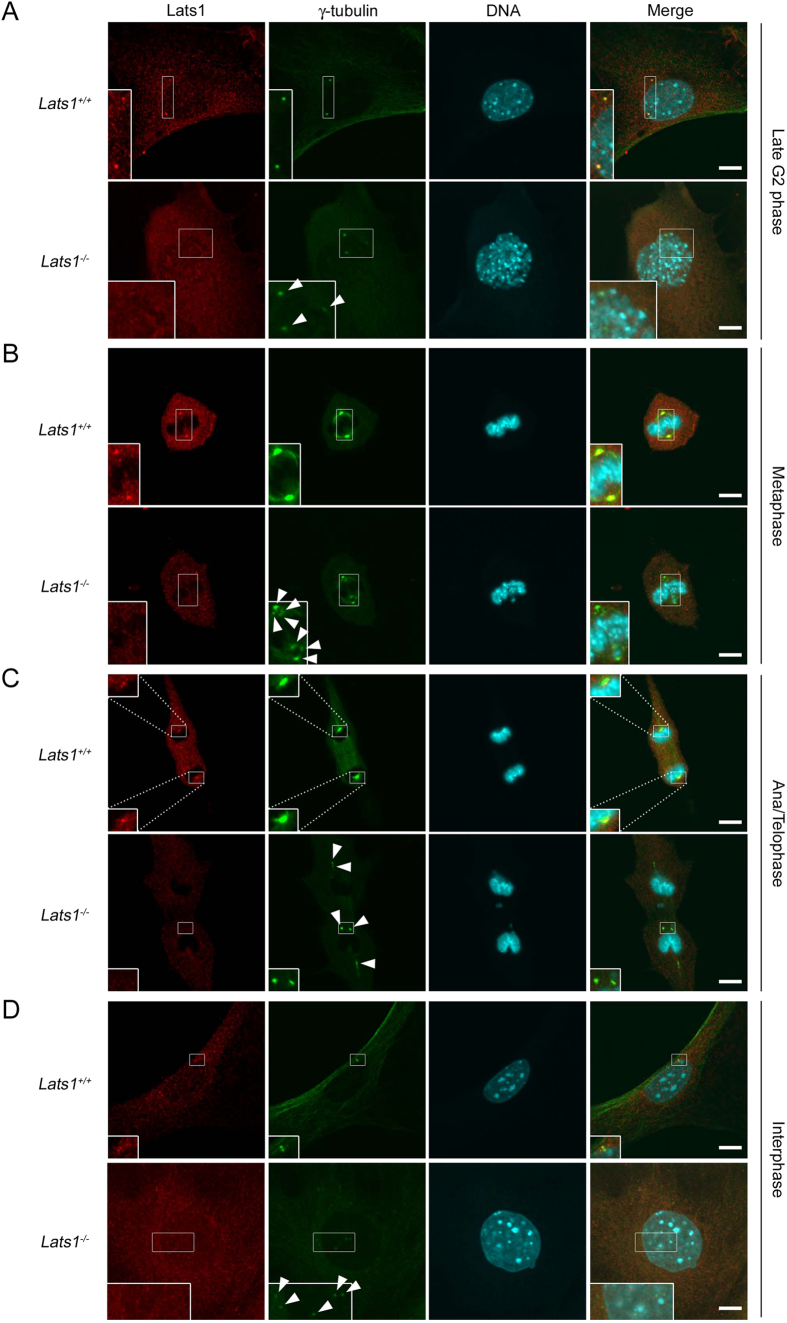
Centrosomal localization of Lats1 during the cell cycle in MEFs. (**A–D**) *Lats1*^+/+^ and *Lats1*^−/−^ MEFs were stained with anti-Lats1 and anti-γ-tubulin antibodies and counterstained with Hoechst 33258 to detect DNA. Late G2, metaphase, ana/telophase, and interphase cells are shown in (**A–D**) respectively. Insets show enlarged images of the centrosomes in the areas outlined by white squares. Arrowheads show supernumerary centrosomes. Scale bar, 10 μm.

Because human LATS1 localizes at the centrosomes during interphase and at the mitotic spindles and spindle poles from metaphase to anaphase^23^, we investigated whether mouse Lats1 actually localizes to the centrosome during the cell cycle. Immunofluorescence analysis revealed that in wild-type (*Lats1*^+/+^) MEFs, Lats1 colocalized with γ-tubulin, a component of the pericentriolar material (PCM) that accumulated substantially at the centrosomes (corresponding to mature centrosomes) during late G2 phase, metaphase, and ana/telophase (Fig. 1A–C, upper panels). During interphase, the Lats1 signal was weak or undetectable at the centrosomes (Fig. 1D, upper panels). To rule out the possibility that these Lats1 signals were experimental artifacts caused by antibody cross-reactions, we examined the centrosomal localization of Lats1 in *Lats1*^−/−^ MEFs. As expected, in *Lats1*^−/−^ MEFs, Lats1 was not detected at the centrosomes or spindle poles at any stage of the cell cycle (Fig. 1A–D, lower panels), indicating that these Lats1 signals were not artificial. These results suggest that mouse Lats1 localizes substantially at the centrosomes during G2/M phase, but faintly or not at all during interphase. This distribution is distinct from that of the human LATS1 protein, which localizes at interphase centrosomes in human cancer cells.

### ***Lats1*** ^**−/−**^ MEFs exhibit centrosome overduplication

**Figure 2:**
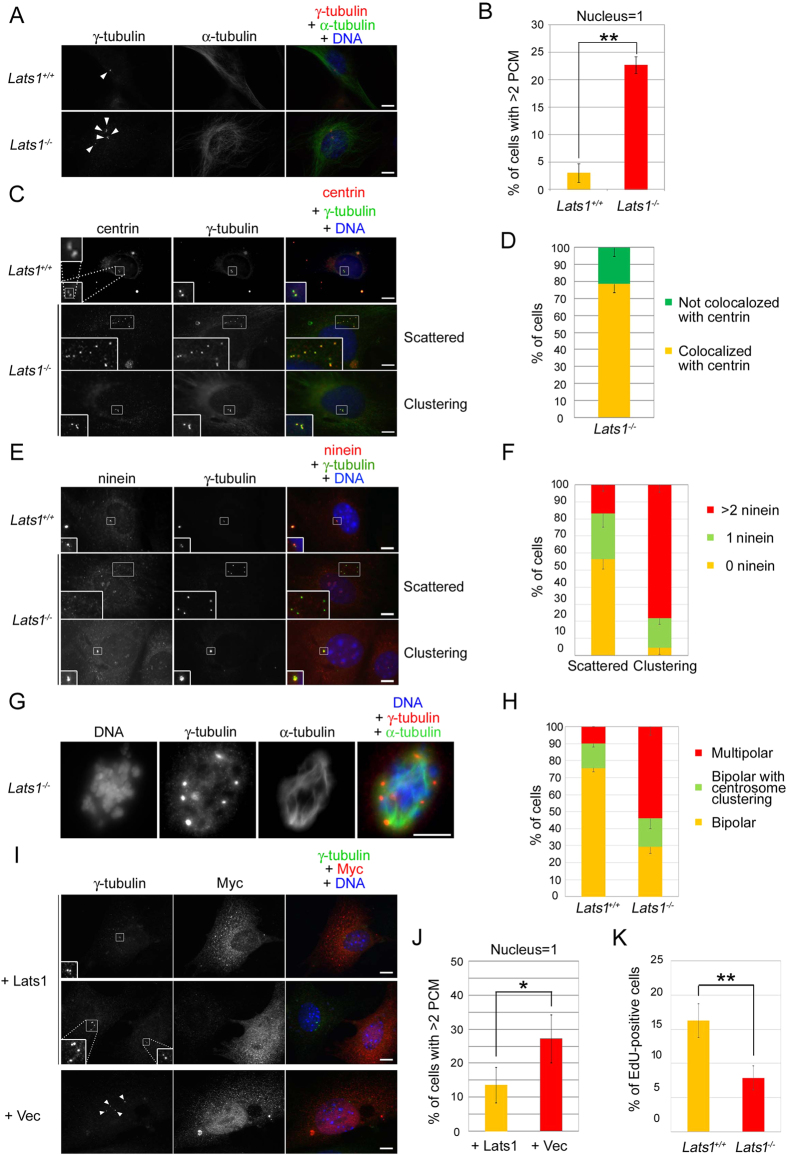
*Lats1*^−/−^ MEFs exhibit centrosome overduplication. (**A**) MEFs were immunostained with the indicated antibodies. Arrowheads indicate γ-tubulin foci. (**B**) MEFs were immunostained as described in A, and mononuclear cells with more than two γ-tubulin foci were counted. Data represent the mean and standard deviation of three independent experiments (n > 100 cells). (**C**) MEFs were immunostained with the indicated antibodies. Insets show enlarged images of the centrosomes in the areas outlined by white squares. (**D**) Frequencies of cells with more than two γ-tubulin foci that colocalized or not with centrin. Data represent the mean and standard deviation of three independent experiments (n > 50 cells). (**E**) MEFs were immunostained with the indicated antibodies. Insets show enlarged images of the centrosomes in the areas outlined by white squares. (**F**) The frequency of cells with the indicated number of ninein foci colocalized with γ-tubulin foci in clustering and scattered centrosomes. Data represent the mean and standard deviation of three independent experiments (scattered; n > 30 cells, clustering; n > 15 cells). (**G**) *Lats1*^−/−^ MEFs were stained with the indicated antibodies. (**H**) MEFs were immunostained as described in E. Cells with multipolar and two different types of bipolar spindles during prophase and metaphase were counted. Data represent mean and standard deviation of three independent experiments (n > 30 cells). (**I**) *Lats1*^−/−^ MEFs were transfected with 6Myc-Lats1 and vector alone. Cells were immunostained with the indicated antibodies. Insets show enlarged images of the centrosomes in the areas outlined by white squares. Arrowheads indicate increased γ-tubulin foci. (**J**) MEFs were immunostained as described in G, and Myc-positive mononuclear cells with more than two γ-tubulin foci were counted. Data represent mean and standard deviation of three independent experiments (n > 40 cells). (**K**) MEFs were treated with 10 μM EdU for 3 h. EdU-positive cells were counted (n > 100 cells). (**A,C,E,G,I**) DNA was visualized by staining with Hoechst 33258. All scale bars indicate 10 μm.

The delocalization of Lats1 from centrosomes in normal MEFs during interphase led us to hypothesize that Lats1 might play an interphase-specific role in centrosome regulation. To test this idea, we investigated the effect of Lats1 deficiency on centrosome duplication by immunofluorescence analysis, using an antibody against γ-tubulin. As shown in Figs 1A,D and 2A, we observed abnormal *Lats1*^−/−^ MEFs with more than two γ-tubulin foci per mononucleated cell. The frequency of these cells was markedly elevated in *Lats1*^−/−^ MEFs (Fig. 2B). To determine whether the generation of excessive γ-tubulin foci was a consequence of centrosome overduplication or fragmentation, we co-stained cells for γ-tubulin and centrin, a component of the centriole (Fig. 2C). The excess γ-tubulin foci were colocalized with centrin foci in the majority of *Lats1*^−/−^ MEFs (78.7%) (Fig. 2D). These results suggest that loss of Lats1 leads to centrosome overduplication. The mechanism of centrosome overduplication is classified into two different categories: one is dysregulation of canonical templated centrosome duplication cycle^1^, and the other is *de novo* formation of centrosome with premature centriole^28^. In general, the centrosomes amplified by canonical duplication cycle tend to form a cluster around the centrosome with a mother centriole, whereas the centrosomes amplified by the *de novo* pathway tend to be dispersed in cytoplasm. Indeed, we found that centrosome overduplication of *Lats1*^−/−^ MEFs occurs by two different processes (Fig. 2C, second panels from top and bottom panels). To examine whether centrosome arises *de novo* in *Lats1*^−/−^ MEFs, the cells were co-immunostained with anti-ninein, a marker of mature centriole, and anti γ-tubulin antibodies. The number of ninein foci colocalized with γ-tubulin foci was counted in cells with clustering or scattered centrosomes. In *Lats1*^−/−^ MEFs with scattered centrosomes, the frequency of cells with 0 or 1 ninein foci were higher than that with >2 ninein foci (Fig. 2E, second panels from top and 2F, left bar graph). On the other hand, in *Lats1*^−/−^ MEFs with clustering centrosomes, the frequency of cells with >2 ninein foci were higher than that with 0 or 1 ninein foci (Fig. 2E, bottom panels and 2F, right bar graph). These results suggest that in *Lats1*^−/−^ MEFs, the clustering centrosomes are generated from a mature centrosome as a template, whereas the scattered centrosomes arise *de novo* before the centriole body becomes mature.

When cells with excessive centrosomes enter M phase, supernumerary centrosomes are clustered and eventually form a bipolar spindle to prevent multipolarity, thereby ensuring successful cell division^29^. We measured the frequencies of bipolar cells with centrosome clustering and multipolar cells in M phase. The frequency of bipolar cells with centrosome clustering during M phase was not increased in *Lats1*^−/−^ MEFs compared with *Lats1*^+/+^ MEFs (Fig. 2H), although *Lats1*^−/−^ MEFs have the excessive centrosomes during interphase (Fig. 2B). Alternatively, the number of *Lats1*^−/−^ cells with multipolar spindle during M phase was 2.4 times more abundant than that of *Lats1*^−/−^ cells with >2 PCM foci during interphase (Fig. 2G,H). These results suggest that spindle multipolarity in *Lats1*^−/−^ MEFs is due to centrosome declustering-dependent or -independent mechanisms, perhaps via spindle pole amplification.

Next, we examined whether enforced expression of Lats1 could rescue the centrosome overduplication in *Lats1*^−/−^ MEFs. The number of γ-tubulin foci in *Lats1*^−/−^ MEFs was markedly decreased by re-expression of 6×Myc-tagged Lats1, relative to expression of the 6Myc-vector alone (Fig. 2I,J), suggesting that Lats1 prevents centrosomal amplification. To rule out the possibility that centrosome overduplication was caused by DNA endoreplication in *Lats1*^−/−^ MEFs during S phase, we examined *de novo* DNA synthesis in *Lats1*^−/−^ MEFs using a 5-ethynyl-2’-deoxyuridine (EdU) incorporation assay. Counting of EdU-positive cells revealed that the proportion of cells that had undergone DNA synthesis was lower in *Lats1*^−/−^ MEFs than in *Lats1*^+/+^ MEFs (Fig. 2K), suggesting that loss of Lats1 causes centrosome overduplication independently of DNA replication. Consistent with the delay of cell cycle, the cell growth rate was also slower in *Lats1*^−/−^ MEFs than in *Lats1*^+/+^ MEFs (Supplementary Fig. S2A online). To rule out the possibility that *Lats1*^−/−^ MEFs underwent apoptosis, apoptotic markers (cleaved caspase-3 and -9) were monitored by western blot analysis (Supplementary Fig. S2B online). The band of cleaved caspase-3 was not detected in *Lats1*^+/+^ and *Lats1*^−/−^ MEFs (top panel, lanes 3 and 4), whereas human cervical cancer cells, HeLa-S3, exhibited the increase of cleaved caspase-3 by UV irradiation (top panel, lane 2). Although in HeLa-S3 cells, pro-caspase-9 (shown by an arrow) was also cleaved and activated by UV irradiation (second panel from top, lanes 1 and 2, arrowheads), the active caspase-9 was not detected in *Lats1*^−/−^ MEFs (second panel from top, lanes 3 and 4). These results suggest that loss of Lats1 leads to cell cycle delay but not apoptosis. Taken together, these results suggest that Lats1 negatively regulates centrosome duplication.

### Loss of Lats1 generates micronuclei and enlarged nuclei

**Figure 3:**
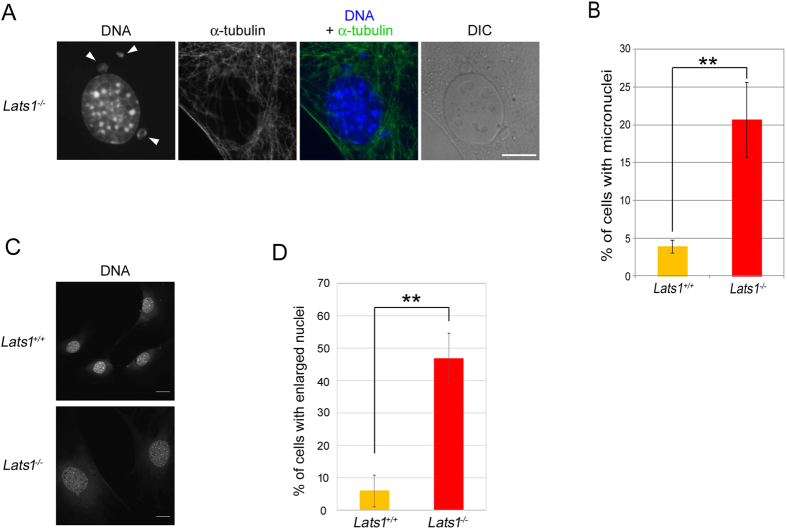
Loss of Lats1 leads to micronuclei and enlarged nuclei. (**A**) Immunofluorescence images show typical *Lats1*^−/−^ MEFs with micronuclei (arrowheads). Cells were stained with anti-α-tubulin antibody and Hoechst 33258 to detect DNA. DIC, differential interference contrast. Scale bar, 20 μm. (**B**) Frequencies of *Lats1*^+/+^ and *Lats1*^−/−^ MEFs with micronuclei. Cells were immunostained as described in A and mononuclear cells with micronuclei were counted. Data represent mean and standard deviation of three independent experiments (n > 100 cells). (**C**) Immunofluorescence images show typical *Lats1*^−/−^ MEFs with large nuclei (arrowheads). Cells were stained as described in A. Scale bar, 20 μm. (**D**) Frequencies of *Lats1*^+/+^ and *Lats1*^−/−^ MEFs with large nuclei. The nuclear area was measured using ImageJ software. Large nuclei were defined as those with areas more than twice that of the average *Lats1*^+/+^ MEFs nucleus. Data represent the mean and standard deviation of three independent experiments (n > 30 cells).

Lats1 deficiency causes cytokinesis failure^25^,^27^,^30^. Indeed, the Lats1-null MEFs exhibited a multinucleated phenotype caused by cytokinesis failure (Supplementary Fig. S3A and B online). Mitotic defects such as centrosome overduplication and cytokinesis failure induce micronuclei and enlarged nuclei through chromosome missegregation. Predictably, *Lats1*^−/−^ MEFs exhibited micronuclei and enlarged nuclei (Fig. 3A,C). Compared with *Lats1*^+/+^ cells, the frequency of mononucleated *Lats1*^−/−^ cells with micronuclei or enlarged nuclei was increased (Fig. 3B,D). These results suggest that loss of Lats1 causes not only centrosome overduplication and cytokinesis defects, but also chromosome missegregation and mitotic slippage.

### Lats1 interacts with Cdc25B

**Figure 4:**
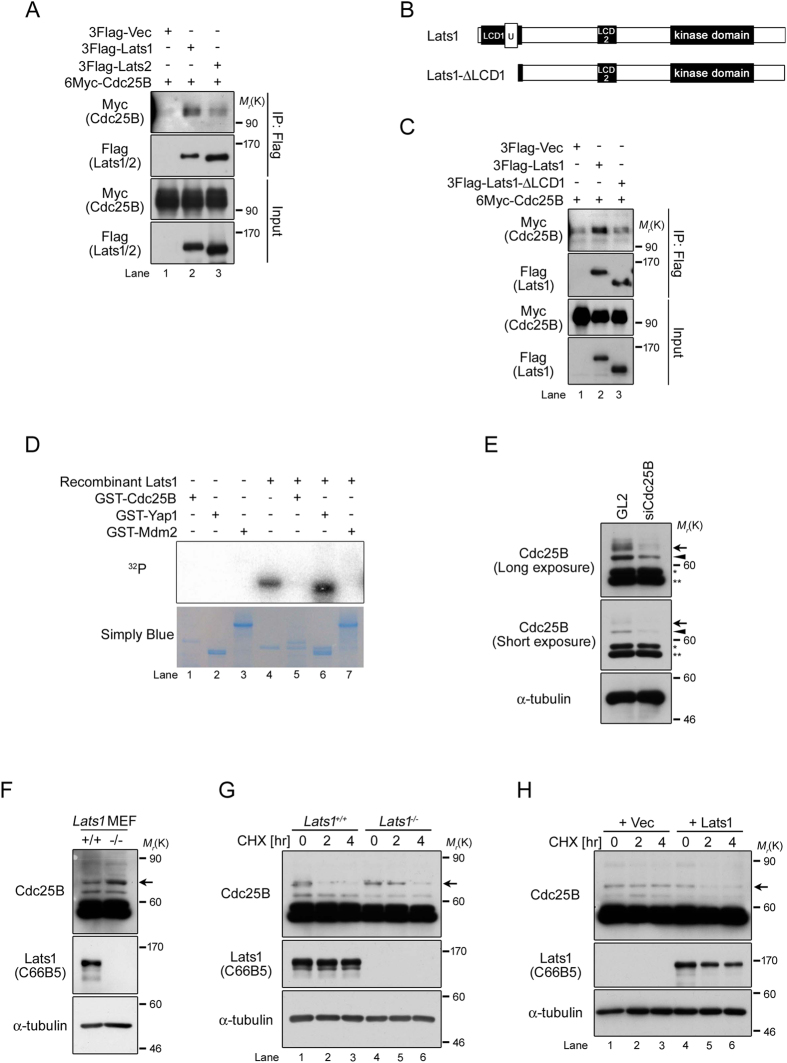
Defect in Lats1 contributes to stabilization of Cdc25B. (**A**) 293T cells were cotransfected with 6Myc-HsCdc25B and 3FLAG-vector (Vec), -HsLats1, or -HsLats2. Proteins were immunoprecipitated with anti-FLAG antibody and detected by western blotting with anti-Myc and anti-FLAG antibodies. *M*_*r*_(K), relative molecular mass (kDa). (**B**) Schematic diagrams of full-length HsLats1 and the HsLats1-ΔLCD1 mutant. U, UBA domain (**C**) 293T cells were cotransfected with 6Myc-HsCdc25B and either 3Flag-Vec, -HsLats1, or -HsLats1-ΔLCD1. Proteins were immunoprecipitated with an anti-FLAG antibody and detected by western blotting with anti-Myc and anti-FLAG antibodies. (**D**) *In vitro* Lats1 kinase assay with GST-HsCdc25B, -MmYap1 (positive control), and HsMdm2 (negative control) in the presence of [γ-^32^P] ATP. SimplyBlue staining was performed to visualize the amounts of loaded proteins. (**E**) Quality check of anti-Cdc25B antibody. *Lats1*^+/+^ MEFs were transfected with siRNA duplex targeting Cdc25B or GL2 (negative control). Lysates were subjected to western blotting with anti-Cdc25B and α-tubulin (loading control) antibodies. Asterisks denote nonspecific bands. Refer to the text for detailed information regarding the bands indicated by the arrow and arrowhead. (**F**) Cdc25B (arrow) and Lats1 in *Lats1*^+/+^ and *Lats1*^−/−^ MEFs were detected by western blot analysis. α-tubulin was used as the loading control. (**G**) *Lats1*^+/+^ and *Lats1*^−/−^ MEFs were treated with cycloheximide (CHX) for the indicated periods. Lysates were probed by western blotting with anti-Cdc25B (arrow) and anti-Lats1 antibodies. α-tubulin was used as the loading control. (**H**) *Lats1*^−/−^ MEFs were transfected with 6Myc-Vec and 6Myc-Lats1. Forty-eight hours after transfection, the cells were treated with cycloheximide (CHX) for the indicated periods. Lysates were detected by western blotting with anti-Cdc25B (arrow) and anti-Lats1 antibodies. α-tubulin was analyzed as the loading control.

Initiation of centriole duplication is triggered by activation of Cdk2, which is regulated by Cdc25 phosphatase family during late G1 phase, followed by phosphorylation of nucleophosmin (NPM)^8^,^31^,^32^. A recent study showed that overexpression of Cdc25B activates Cdk2, resulting in centrosome overduplication during interphase^16^. To elucidate the function of Lats1 in centrosome duplication, we focused on exploring the molecular relationship of Lats1 to Cdc25B and Cdk2 in some regulators of centrosome duplication. Because Lats1 does not interact with Cdk2 under non-stimulated conditions^33^, we first examined the interactions between Cdc25B and Lats1 or Lats2. To this end, we cotransfected human embryonic kidney 293T cells with 6×Myc-tagged Cdc25B and 3×FLAG-tagged Lats1 or Lats2, followed by immunoprecipitation and western blotting. Cdc25B associated more strongly with Lats1 than Lats2 (Fig. 4A). Previously, we showed that disruption of the N-terminal region of Lats1 resulted in centrosomal overduplication^27^, indicating that the N-terminus of Lats1 may be important for regulation of centrosome duplication. To determine whether the N-terminal region of Lats1 is required for the interaction with Cdc25B, we constructed a Lats1 deletion mutant lacking the N-terminus (amino acids 1–141), including the UBA (ubiquitin-associated) domain (Fig. 4B, Lats1-ΔLCD1). An immunoprecipitation assay revealed that Cdc25B interacted much more weakly with Lats1-ΔLCD1 than with full-length Lats1 (Fig. 4C), suggesting that the N-terminal region (including the LCD1 and UBA domains) of Lats1 is essential for binding to Cdc25B. Next, we investigated whether Lats1 phosphorylates Cdc25B. An *in vitro* kinase assay revealed that Cdc25B was not phosphorylated by Lats1; Lats1 did phosphorylate Yap (a positive control), but not MDM2 (a negative control) (Fig. 4D). These results suggest that Lats1, rather than Lats2, serves as a regulator of Cdc25B in a manner that is independent of kinase activity.

### A defect in Lats1 contributes to stabilization of Cdc25B

Five major splicing variants of human Cdc25B have been identified to date^34^. An anti-Cdc25B antibody detected at least four bands, including two major bands (50–59 kDa) and two minor bands (60–80 kDa) in asynchronously growing wild-type MEFs (Fig. 4E, lane 1). In *Lats1*^+/+^ MEFs, two minor bands (arrow and arrowhead) were attenuated by siRNA-mediated knockdown of Cdc25B, whereas two major bands (asterisks) did not change (Fig. 4E, lane 2). Moreover, western blot analysis using serial dilution of the extract from Cdc25B knockdown cells revealed that two minor bands were increased in a dose-dependent manner (Supplementary Fig. S4A online, arrow and arrowhead). However, because only the slower-migrating of the two minor bands was successfully attenuated using other siRNAs against Cdc25 (siCdc25B-A, B and C), we concluded that endogenous Cdc25B protein in MEFs is primarily reflected by this slower-migrating band (Supplementary Fig. S4B online, arrow).

When we assessed the expression levels of Cdc25B protein in *Lats1*^+/+^ and *Lats1*^−/−^ MEFs by western blotting, Cdc25B was present at higher levels in *Lats1*^−/−^ MEFs than in *Lats1*^+/+^ MEFs (Fig. 4F). To compare the stability of Cdc25B protein in *Lats1*^+/+^ and *Lats1*^−/−^ MEFs, we treated the cells with cycloheximide (CHX) to inhibit *de novo* protein synthesis. Cdc25B protein levels in *Lats1*^+/+^ MEFs were significantly lower 2 hours after CHX treatment (Fig. 4G, lane 2); by contrast, Cdc25B protein levels in *Lats1*^−/−^ MEFs remained stable for 2 hours after CHX treatment (Fig. 4G, lane 5). Notably, stabilization of Cdc25B by Lats1 deficiency was abolished by ectopic overexpression of Lats1 in *Lats1*^−/−^ MEFs (Fig. 4H). These results suggest that loss of Lats1 inhibits the degradation of Cdc25B protein.

### Cdk2 activity evokes centrosome overduplication in ***Lats1*** ^**−/−**^ MEFs

**Figure 5:**
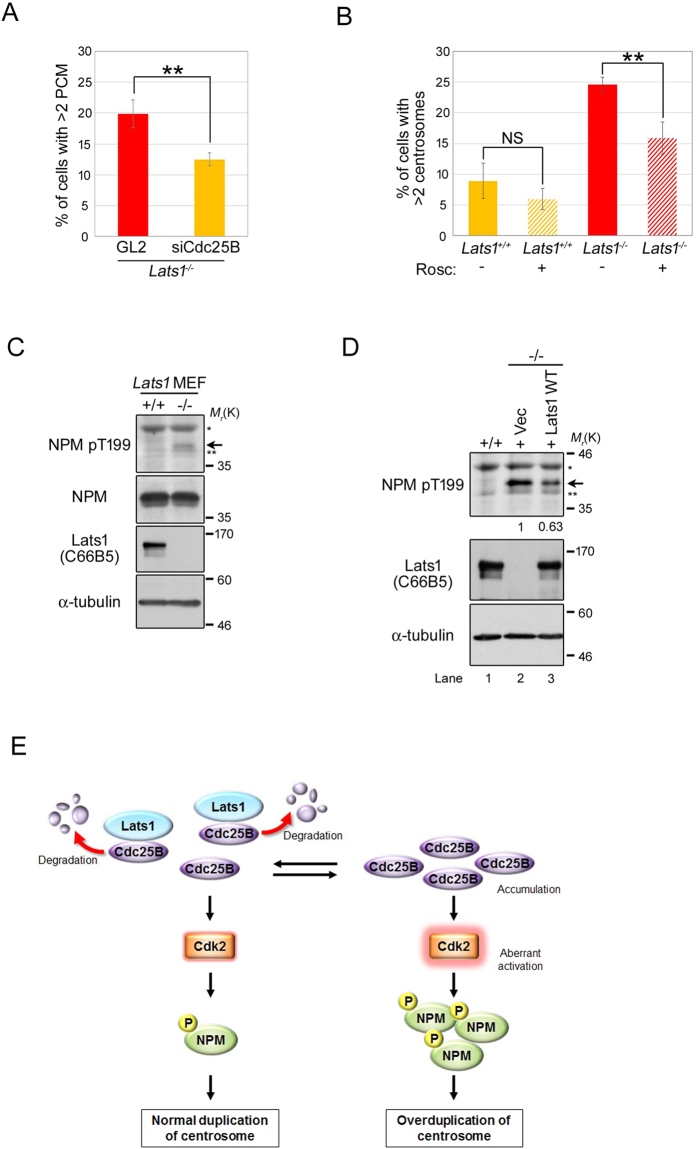
Dysregulation of the Cdc25B-Cdk2-NPM axis contributes to centrosome overduplication in *Lats1*^−/−^ MEFs. (**A**) *Lats1*^−/−^ MEFs transfected with Cdc25B siRNA were immunostained with an anti-γ-tubulin antibody and counterstained with Hoechst 33258 to visualize DNA. GL2 was used as a negative control. The percentages of mononuclear cells with more than two γ-tubulin foci are shown. Data represent mean and standard deviation of three independent experiments (n > 100 cells). (**B**) *Lats1*^+/+^ and *Lats1*^−/−^ MEFs were treated with or without 180 μM roscovitine (Rosc) for 24 hr. The cells were stained with anti-centrin and anti-γ-tubulin antibodies. The percentages of mononuclear cells with more than two centrosomes are shown (n > 100 cells). (**C**) *Lats1*^+/+^ and *Lats1*^−/−^ MEFs were immunoblotted with anti-NPM-pT199, anti-NPM, and anti-Lats1 antibodies. α-tubulin was analyzed as the loading control. (**D**) *Lats1*^−/−^ MEFs were transfected with 6Myc-Vec, -Lats1-WT, and -Lats1-KD. NPM-pT199 was detected by western blotting. Signal intensity was measured using ImageJ software and normalized to α-tubulin. (**E**) A model for regulation of centrosome duplication by Lats1.

Because overexpression of Cdc25B causes formation of excess centrosomes through Cdk2 activation^16^, we hypothesized that protein stabilization and upregulation of Cdc25B followed by Cdk2 activation would induce centrosome overduplication in *Lats1*^−/−^ MEFs. To test this idea, we examined the effect of Cdc25B-mediated Cdk2 activation on centrosome number in *Lats1*^−/−^ MEFs. Immunostaining with a γ-tubulin antibody revealed that depletion of Cdc25B by siRNA decreased the number of excess γ-tubulin foci in *Lats1*^−/−^ MEFs (Fig. 5A). Moreover, overduplication of the centrosome in *Lats1*^−/−^ MEFs was rescued by treatment with a Cdk inhibitor, roscovitine (Rosc), although the centrosome number in *Lats1*^+/+^ MEFs was not altered in the presence or absence of Rosc (Fig. 5B). These results suggest that Cdc25B accumulation and activation of Cdk2 cause centrosome overduplication in *Lats1*^−/−^ MEFs.

Thr-199 (T199) of NPM is a target of phosphorylation by Cdk2, which initiates centrosome duplication by promoting centriole disengagement^32^,^35^. Hence, we next examined the impact of Lats1 deficiency on the phosphorylation level of NPM-T199. Levels of NPM pT199 were higher in *Lats1*^−/−^ MEFs than in wild-type MEFs (Fig. 5C, arrow). The increased phosphorylation of NPM-T199 in *Lats1*^−/−^ MEFs was suppressed by ectopic expression of wild-type Lats1 (Fig. 5D, lane 3). These results suggest that Lats1 negatively regulates Cdk2 activity toward NPM by inhibiting Cdc25B.

Based on these findings, we propose that Lats1 functions as a negative regulator of the Cdc25B-Cdk2 axis by binding directly to Cdc25B, thereby preventing overduplication or untimely duplication of centrosomes through phosphorylation of NPM (Fig. 5E).

## Discussion

In a previous study, we generated *Lats1*^*ΔN/ΔN*^ mice by disrupting the region encoding the N-terminus of Lats1^27^. MEFs established from *Lats1*^*ΔN/ΔN*^ mice endogenously express an N-terminally truncated Lats1 protein, whose kinase activity is retained at least *in vitro*. These cells also exhibit centrosome overduplication. Here, we generated Lats1-null mice and derivative cells (*Lats1*^−/−^ MEFs). Using *Lats1*^−/−^ MEFs, we demonstrated that Lats1 contributes to the suppression of centrosome amplification by limiting the protein level of Cdc25B (Fig. 5E). Importantly, the N-terminal region of Lats1, including the LCD1 and UBA domain, is required for the binding of Cdc25B, although Lats1 does not directly phosphorylate Cdc25B (Fig. 4C,D). Because the UBA domain associates with the polyubiquitin chain of some proteins that are destined to be degraded, and recruits them to the proteasome^36^, it is possible that the UBA domain of Lats1 interacts with ubiquitinated Cdc25B, promoting protein degradation of Cdc25B by efficiently recruiting it to the proteasome. In support of this idea, *Lats1*^*ΔN/ΔN*^ MEFs expressing UBA domain-truncated Lats1 protein also exhibit centrosome overduplication^27^, potentially due to loss of the interaction between Lats1 and Cdc25B (Fig. 4C). Moreover, we found that accumulation of Cdc25B protein due to Lats1 deficiency causes aberrant activation of Cdk2 and subsequently promotes the phosphorylation of NPM (Fig. 5B,C). These results suggest that Lats1 plays an important role in the licensing of centrosome duplication by fine-tuning the phosphorylation state of NPM via the Cdc25B-Cdk2 axis.

Lats1 and Lats2 are classified as members of the Dbf2 kinase family, which includes nuclear Dbf2-related proteins 1 and 2 (NDR1 and NDR2)^37^. Previous work showed that overexpression of NDR1 and NDR2, but not Lats1 and Lats2, causes centrosome amplification in U2-OS cells^38^, suggesting that Lats1 and Lats2 are dispensable for the promotion of centrosome duplication. Consistent with this, our results suggest that Lats proteins, unlike NDR proteins, function as suppressors of centrosome duplication, especially overduplication.

Although centrosome duplication is induced during S phase, the majority of Lats1 localizes in the cytoplasm and nucleus during this phase, with little or no Lats1 detectable at the centrosome^27^ (Fig. 1D). Fluctuations in the total Cdc25B protein level in cells affect the abundance of centrosomal Cdc25B and the subsequent accumulation of centrosomal proteins such as centrin, ultimately affecting centrosome number^16^. Therefore, one possibility is that cytoplasmic or nuclear Lats1 may influence the level of Cdc25B at the centrosome by regulating the total Cdc25B level. Another possibility is that the abundance of centrosomal Lats1 itself may be stringently regulated by cellular degradation machinery, such as the proteasome, in the vicinity of the centrosome during interphase, in order to prevent inappropriate inhibition of centrosome duplication. On the other hand, between late G2 and M phase, Lats1 also localize to the centrosomes and the spindle poles of mouse cells (Fig. 1A–C), consistent with previous reports regarding the subcellular localization of Lats1 in human cells^23^,^24^. Salvador (also known as WW45 and hSav) and Mst2, which are core components of the Hippo pathway, also localize at the centrosome; together with Nek2A kinase, these proteins cooperatively regulate the disjunction of centrosomes at mitotic entry^39^, Lats1 might colocalize with Salvador and/or Mst2 at the mitotic centrosome and coordinate the functions of these proteins in centrosome disjunction. Moreover, Lats1 appears to be phosphorylated by Cdk1/cyclin B at the spindle poles during mitosis^40^. However, the biological function of Lats1 at the mitotic centrosome remains unclear.

In our previous study, *Lats2*^−/−^ MEFs exhibited centrosome fragmentation (excess γ-tubulin foci without centriole amplification) but not centrosome overduplication (excess centrin foci with centriole amplification)^21^. Here, we showed that Cdc25B interacts more weakly with Lats2 than Lats1 (Fig. 4A). Although the N-terminal regions of Lats1 and Lats2 have a relatively high conserved region, LCD1, which is required for binding to Cdc25B, the overall sequence similarity between their N-terminal regions is very low. Therefore, it is thought that Cdc25B binds to Lats1 and Lats2 via different mechanisms, leading to the dramatically different effects of Lats1 and Lats2 deficiency on centrosome integrity. The Lats1-null knockout mice generated in this study, as well as other Lats1-deficient mice generated by disrupting different genome regions of *Lats1*^26^,^27^, were born normally but exhibited growth retardation and smaller body size (i.e., dwarfism), whereas Lats2 deficiency is lethal before embryonic day 12^21^. Primordial dwarfism, a human disorder that involves an extreme reduction in growth and body size, is associated with loss-of-function mutations in genes coding several centrosomal proteins, including core components involved in centrosome duplication (e.g., PCNT, CPAP, and CEP152), the pre-replicative complex (e.g., ORC1, ORC4, ORC6, CDT1, and CDC6), and the DNA damage checkpoint (e.g., ATR and MCPH1)^41^. Notably, in *Lats1*^−/−^ MEFs, the proportion of cells that had undergone DNA synthesis was reduced, despite the overduplication of centrosomes (Fig. 2I). Moreover, Lats1 interacts with CDK2 and inhibits its kinase activity toward BRCA2 on S3291 downstream of the ATR-RASSF1A-MST2 axis, thereby preventing replication fork instability in response to replication stress^42^. Therefore, centrosomal Lats1 seems to coordinately regulate precise progression of DNA replication and centrosome during S phase, which might explain its role in disorders associated with body growth failure, such as dwarfism, although cytoplasmic or nuclear Lats kinases are known to suppress overgrowth of certain organs (e.g., liver) by inhibiting Yap and Taz in the Hippo pathway.

As shown in Fig. 2C, individual γ-tubulin foci in *Lats1*^−/−^ MEFs do not appear to colocalize with two centrin foci but rather with only one centrin focus, suggesting that each γ-tubulin focus contains only one centriole (these have been called “singlet centrioles” in the literature). In *Lats1*^+/+^ MEFs, centrin-staining area in each γ-tubulin focus was almost completely merged with γ-tubulin-staining area, although we could not clearly discriminate the number (one or two) of centrin foci in each γ-tubulin focus (Fig. 2C, top panels). This result does not show that γ-tubulin focus contains only one centriole, suggesting that this centrosome is not a “singlet centriole”. Likewise, the scattered centrosomes in *Lats1*^−/−^ MEFs do not seem to be singlet centrioles (Fig. 2C, second panels from top). In contrast, the clustering centrosomes of *Lats1*^−/−^ MEFs seem to be singlet centrioles (Fig. 2C, bottom panels). Therefore, singlet centrioles may be a unique phenotype of clustering centrosomes of *Lats1*^−/−^ MEFs. The proximity of these singlet centrioles to one another in the clustering centrosomes of *Lats1*^−/−^ MEFs suggests that premature centriole disengagement may be occurring, with consequent “templated” centrosome overduplication. These results suggest that in *Lats1*^−/−^ MEFs the scattered centrosomes arise from *de novo* overduplication, whereas the clustering centrosomes arise from canonical centrosome overduplication with premature disengagement; however, since it seems that γ-tubulin foci may colocalize with only single centrioles, cells with >2 γ-tubulin foci (specifically, cells with 4 foci) may not actually have supernumerary centrosomes. Thus, our claim about the extent of overduplication in the clustered centrosomes may be overstated.

Centrosome overduplication potentially induces chromosome fragmentation and missegregation, followed by formation of micronuclei^43^. Because micronuclei can indicate chromosomal instability, they have been used as a tool to understand the pathogenesis and the malignancy in human tumor cells^43^. We also found that the proportion of abnormal cells with micronuclei is elevated in *Lats1*^−/−^ MEFs, suggesting that in MEFs, Lats1 plays an important role in chromosomal instability through the centrosome overduplication. Indeed, overexpression of Cdc25B and downregulation or mutation of Lats1 and Lats2 are frequently observed in various human cancers^11^,^37^,^44^, and loss of the N-terminus (LCD1) of Lats1 can cause tumor formation in nude mice^19^. Thus, in human malignant tumors, deficiency in Lats1 and/or Lats2 might promote overexpression of Cdc25B, leading to centrosome overduplication and, ultimately, to cancer progression. However, even though human Lats1 can interact with human Cdc25B (Fig. 4A), it remains unclear whether Lats1 is involved in centrosome duplication during interphase in human normal and/or cancer cells. Therefore, further studies are needed to determine whether human Lats1 contributes to centrosomal integrity, including duplication and its licensing, as in the case of mouse Lats1.

## Methods

### Generation of the *Lats1* targeted allele

The mouse *Lats1* gene was disrupted by replacing part of exon 5 (E5) (amino acids 684–853), which encodes a large part of the kinase domain (Supplementary Fig. S1A online). An ES cell (clone #40) identified as harboring a correctly targeted construct was injected into 8-cell stage embryos, which were transferred to pseudopregnant females to generate chimeric mice. PCR analysis using primers B and C (Supplementary Fig. S1A online) was used to identify *bona fide* chimeric mice (Supplementary Fig. S1B online, #40-2, -5, -12, and -13). Next, these chimeric mice were bred with C57BL/6 mice to produce F1 heterozygotes. Homozygous C57BL/6 mice [*Lats1*^−/−^ (Accession No. CDB0938K: http://www.clst.riken.jp/arg/mutant%20mice%20list.html)] were obtained by intercrossing the heterozygous offspring. Mouse genotypes were confirmed by PCR analysis of genomic DNA derived from the tails of the offspring using primer pairs A (primer A and C) and B (primer B and C) (Supplementary Fig. S1C online).

### Cell culture

Primary MEFs were isolated from mouse embryos at 12.5 days post coitum, as described previously^21^. Cultured MEFs were maintained in MEF medium, consisting of Dulbecco’s modified Eagle’s medium (DMEM, Sigma, St. Louis, MO) supplemented with inactivated 10% fetal bovine serum (FBS, Hyclone, Logan, UT), 100 U/mL penicillin, 100 μg/mL streptomycin, and 55 μM β-mercaptoethanol. Human embryonic kidney 293T cells were maintained in DMEM supplemented with 10% FBS, 100 U/mL penicillin, and 100 μg/mL streptomycin. HeLa-S3 cells were maintained in DMEM supplemented with 5% FBS, 100 U/mL penicillin, and 100 μg/mL streptomycin.

### Drug treatment

Cells were treated with 180 μM roscovitine (Calbiochem, La Jolla, CA, USA), a Cdk inhibitor, together with 2.5 mM thymidine (Nacalai Tesque, Kyoto, Japan) for 24 h. Cells were incubated with 50 μg/ml cycloheximide (Nacalai Tesque), a protein synthesis inhibitor, for 0, 2, or 4 h.

### EdU incorporation assay

Cells were treated with 10 μM EdU for 3 h, and then fixed in cold methanol for 20 min at −20 °C. Fixed cells were detected by EdU staining using the Click-iT Plus EdU Alexa Fluor 594 Imaging kit (Invitrogen, Carlsbad, CA, USA).

### UV irradiation

For UV irradiation of cells using a germinal lamp (Stratalinker 2400, Stratagene, La Jolla, CA) at 50 J/m^2^, the culture medium was removed, and then cells were washed with PBS without calcium and magnesium [PBS(−)]. After UV irradiation, the culture medium was returned to the cell plate and incubated at 37 °C for 18 h.

### Transfection and siRNA

siRNA duplexes and plasmid DNA were transfected using Lipofectamine 2000 or Lipofectamine with PLUS Reagent, respectively (Invitrogen). Four kinds of siRNA duplex against Cdc25B were purchased [siCdc25B from Santa Cruz Biotechnology (Dallas, TX, USA), and siCdc25B-A, -B, and -C were purchased from OriGene (Rockville, MD, USA)]. GL2 is siRNA duplex targeting firefly luciferase as negative control.

### Plasmids

6×Myc-mouse (Mm) Lats1 and GST-MmYap1 were described previously^19^,^27^. Human (Hs) Lats1 and HsLats2 cDNAs were subcloned into the *Asc*I and *Not*I sites of the p3FLAG vector, a modified version of p3×FLAG-CMV-7.1 vector. HsLats1-ΔLCD1 (aa 142–1130) was generated by PCR using pCMV6-myc-HsLats1^19^ as a template, and subcloned into the p3FLAG vector. The coding regions of HsMdm2 was cloned by PCR from human myometrium cDNA libraries, and HsCdc25B was chemically synthesized (GenScript, Piscataway, NJ, USA). They were subcloned into the *Asc*I and *Not*I sites of pGST6P, a GST-fused protein expression vector, and pCMV6-myc. All amplified nucleotide sequences were confirmed by DNA sequencing.

### Antibodies

Monoclonal (mAb) and polyclonal (pAb) antibodies against the following proteins were used: FLAG-tag pAb, centrin-2 pAb, γ-Tubulin pAb/mAb, and α-Tubulin mAb (all from Sigma); Lats1 rabbit mAb (C66B5), Cdc25B pAb, and cleaved caspase-3 pAb (Cell Signaling, Beverly, MA, USA); Lats1 pAb (Bethyl Laboratories, Montgomery, TX, USA); and GAPDH mAb, Myc-tag mAb (PL14), and caspase-9 mAb (MBL, Nagoya, Japan); ninein pAb (Abcam, Cambridge, UK).

### Immunoprecipitation

Collected cell pellets were resuspended in modified TNE250 lysis buffer (10 mM Tris-HCl [pH 8.0], 250 mM NaCl, 1 mM EDTA, 0.25% NP-40, 1 mM dithiothreitol, 2 mM benzamidine) supplemented with 100 μg/mL PMSF, 1 μg/mL aprotinin, 10 μg/mL leupeptin, 1 μg/mL pepstatin A, 1 mM NaF, 1 mM Na_3_VO_4_, 10 mM β-glycerophosphate, and 100 ng/mL okadaic acid (OA) at 4 °C for 30 min. After centrifugation, the supernatants were pre-cleared with 50% protein A-Sepharose at 4°C for 1 h. The cleared lysates were incubated with 2 μg of anti-FLAG pAb at 4 °C for 2 h. Then, the immune complexes were harvested by the addition of 30 μl of 50% protein A-Sepharose for 2 h, and then washed in NETN150 buffer (20 mM Tris-HCl, pH7.5, 150 mM NaCl, 1 mM EDTA, 0.5% NP-40) supplemented with 10 μg/mL PMSF, 1 μg/mL aprotinin, 10 μg/mL leupeptin, 1 μg/mL pepstatin A, 1 mM NaF, 1 mM Na_3_VO_4_, and 10 mM β-glycerophosphate.

### Western blot analysis

Whole-cell lysates were prepared as previously described^19^,^21^. Briefly, cells were lysed in modified TNE250 lysis buffer (10 mM Tris-HCl [pH 8.0], 250 mM NaCl, 1 mM EDTA, 0.25% NP-40, 1 mM dithiothreitol, 2 mM benzamidine) or RIPA buffer (10 mM Tris-HCl [pH 7.5], 150 mM NaCl, 1 mM EDTA, 1% NP-40, 1 mM dithiothreitol, 0.1% SDS, 0.1% deoxycholate) supplemented with 100 μg/mL PMSF, 1 μg/mL aprotinin, 10 μg/mL leupeptin, 1 μg/mL pepstatin A, 1 mM NaF, 1 mM Na_3_VO_4_, 10 mM β-glycerophosphate, and 100 g/mL OA at 4 °C for 30 min. After centrifugation, cleared lysates were subjected to IP and SDS-PAGE. Proteins were resolved by SDS-PAGE and transferred to PVDF membranes, followed by western blotting with the indicated antibodies in TBST with 5% nonfat milk after blocking. Dot-blot analysis was performed as previously described^19^.

### *In vitro* kinase assay

Recombinant HsLats1 kinase (Carna Biosciences, Hyogo, Japan) was incubated with GST-fused HsCdc25B, -HsMdm2, or -MmYap1^27^ in Lats1-kinase buffer (20 mM PIPES [pH 6.8], 4 mM MnCl_2_, 1 mM DTT, 1 mM NaF, 1 mM Na_3_VO_4_) containing 20 μM ATP and 10 μCi [γ-^32^P] ATP. Reactions were resolved by SDS-PAGE followed by autoradiography. The mini-gel was stained using SimplyBlue SafeStain (Invitrogen).

### Immunofluorescence staining

Indirect immunofluorescence staining was performed as previously described^21^. In brief, exponentially growing MEFs were plated on coverslips and consecutively fixed at room temperature for 10 min each in 4% formaldehyde in PBS, 0.1% Triton X-100 in PBS(−), and 0.05% Tween-20 in PBS. To visualize centrosomes and microtubules, cells were incubated with antibodies against α-tubulin and γ-tubulin, followed by incubation with Alexa Fluor 488- and 594-conjugated anti-rabbit/mouse IgG (Molecular Probes, Eugene, OR) in TBST (100 mM Tris-Cl [pH 7.5], 150 mM NaCl, 0.05% Tween-20) containing 5% FBS. For staining of centrin and Lats1, cells were fixed for 20 min at −20 °C in cold-methanol/acetone (1:1) and cold methanol, respectively, and then washed with PBS(−). The fixed cells were stained with anti-centrin or anti-Lats1 (C66B5) antibody in TBST containing 5% BSA. DNA was stained with Hoechst 33258 (Sigma). Cells were observed under a BX51 microscope (Olympus, Tokyo, Japan).

### Statistical analysis

Statistical analysis was performed using Excel. Error bars for all data represent the standard deviation from the mean. *P*-values were calculated using Student’s *t*-tests. *P < 0.05 and **P < 0.01.

### Ethical permission

All animal experiments were approved by the Animal Care and Use Committee of the Research Institute for Microbial Diseases, Osaka University, Japan (#Biken-AP-H24-16-0). The methods were carried out in accordance with the approved guidelines.

## Additional Information

**How to cite this article**: Mukai, S. *et al.* Lats1 suppresses centrosome overduplication by modulating the stability of Cdc25B. *Sci. Rep.* **5**, 16173; doi: 10.1038/srep16173 (2015).

## Supplementary Material

Supplementary Information
