# Supplementary material for: Lats1 suppresses centrosome overduplication by modulating the stability of Cdc25B
Source: Sci Rep. 2015 Nov 4;5:16173. doi: 10.1038/srep16173 (PMC4632022; doi:10.1038/srep16173)
Supplement: Supplementary Information [file srep16173-s1.pdf]

Supplementary information

Lats1 suppresses centrosome overduplication by modulating the stability of Cdc25B

Satomi Mukai[1], Norikazu Yabuta[1]§, Kaori Yoshida[1], Ayumi Okamoto[1], Daisaku Miura[2], Yasuhide Furuta[3,4], Takaya Abe[3], and Hiroshi Nojima[1]

[1]Department of Molecular Genetics Research Institute for Microbial Diseases, Osaka University, 3-1 Yamadaoka, Suita City, Osaka 565-0871, Japan; [2]Department of Pharmacy, Hyogo University of Health Sciences, Kobe, Japan; [3]Animal Resource Development Unit and [4]Genetic Engineering Team, RIKEN Center for Life Science Technologies, 2-2-3 Minatojima-minamimachi, Chuo-ku, Kobe, Hyogo 650-0047, Japan.

§ Correspondence to N. Yabuta

Phone: +81-6-6875-3980, Fax: +81-6-6875-5192

E-mail: nyabuta@biken.osaka-u.ac.jp

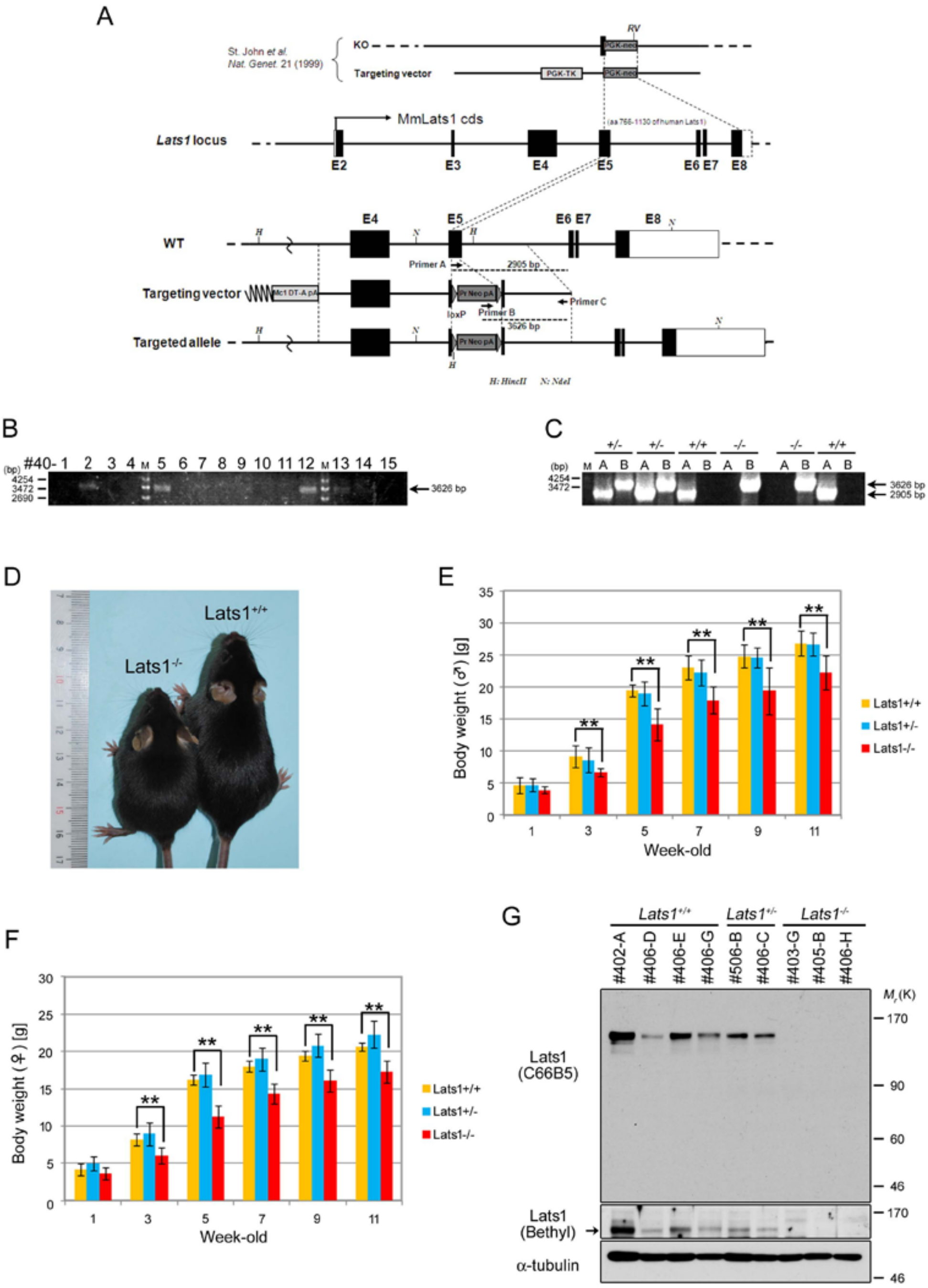

Figure S1. Generation of *Lats1*$^{-/-}$ knockout mice and establishment of *Lats1*$^{-/-}$ MEFs.
(A) Schematic diagrams of the *Lats1* locus, targeting vector, and targeted allele. The *Lats1* gene was disrupted by replacing a part of the fourth coding exon (E5) with the neomycin selection cassette in the targeting vector and targeted allele. Black boxes indicate coding exons. Arrows show PCR primer positions. The top diagram shows the targeting strategy for another *Lats1*-knockout mouse reported by St. John et al. (Nat. Genet., 21, 1999). WT, wild-type; KO, knockout. (B) Chimeric mice were identified by PCR analysis using primers B and C (shown in [A]). Mutant products (3626 bp) were amplified from genomic DNA of #40-2, -5, -12, and -13. M, size marker. (C) Genomic PCR analysis of tails of offspring obtained from heterozygote intercrosses. Lane A, primers A and C (shown in [A]) were used to amplify WT products (2905 bp); lane B, primers B and C (shown in [A]) were used to amplify mutated products (3625bp). M, size marker. (D) Representative picture of *Lats1*$^{-/-}$ and *Lats1*$^{+/+}$ mice. (E, F) Body weights of *Lats1*$^{+/+}$, *Lats1*$^{+/-}$, and *Lats1*$^{-/-}$ male (E) and female mice (F) from 1 to 11 weeks after birth. Data represent average body weights of more than three offspring for each genotype. (G) Western blot analysis using cell lysates from *Lats1*$^{+/+}$, *Lats1*$^{+/-}$, and *Lats1*$^{-/-}$ MEFs. Lats1 proteins were recognized by an anti-Lats1 monoclonal antibody (C66B5) against the N-terminus and an anti-Lats1 polyclonal antibody (Bethyl) against the C-terminus. M_r(K), relative molecular mass (kDa).

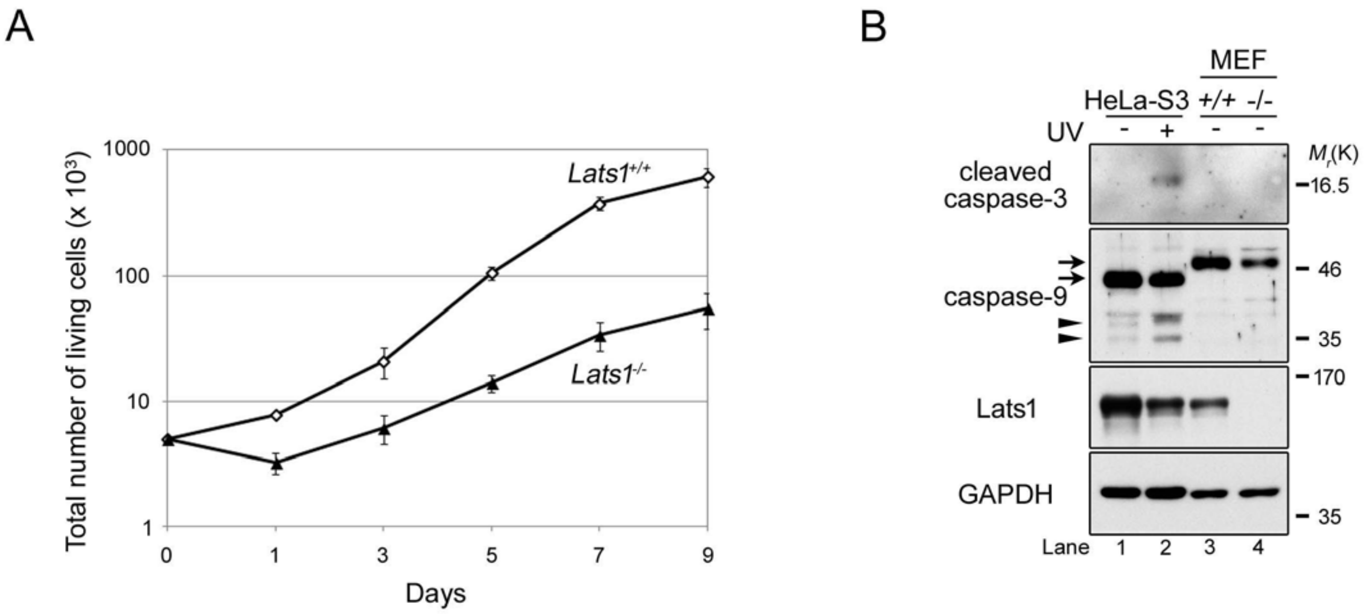

Figure S2. ***Lats1*** $^{-/-}$ **MEFs exhibit cell cycle delay.**

(A) Cell growth curves of immortalized *Lats1* $^{+/+}$ (open rhombus) and *Lats1* $^{-/-}$ (closed triangle) MEFs. Means and standard deviations were derived from three individual experiments. (B) Western blot analysis using cell lysates from HeLa-S3 cells with or without UV irradiation, *Lats1* $^{+/+}$, and *Lats1* $^{-/-}$ MEFs. Cleaved caspase-3, caspase-9 (arrows), active caspase-9 (arrowheads), and Lats1 were detected. GAPDH was analyzed as the loading control.

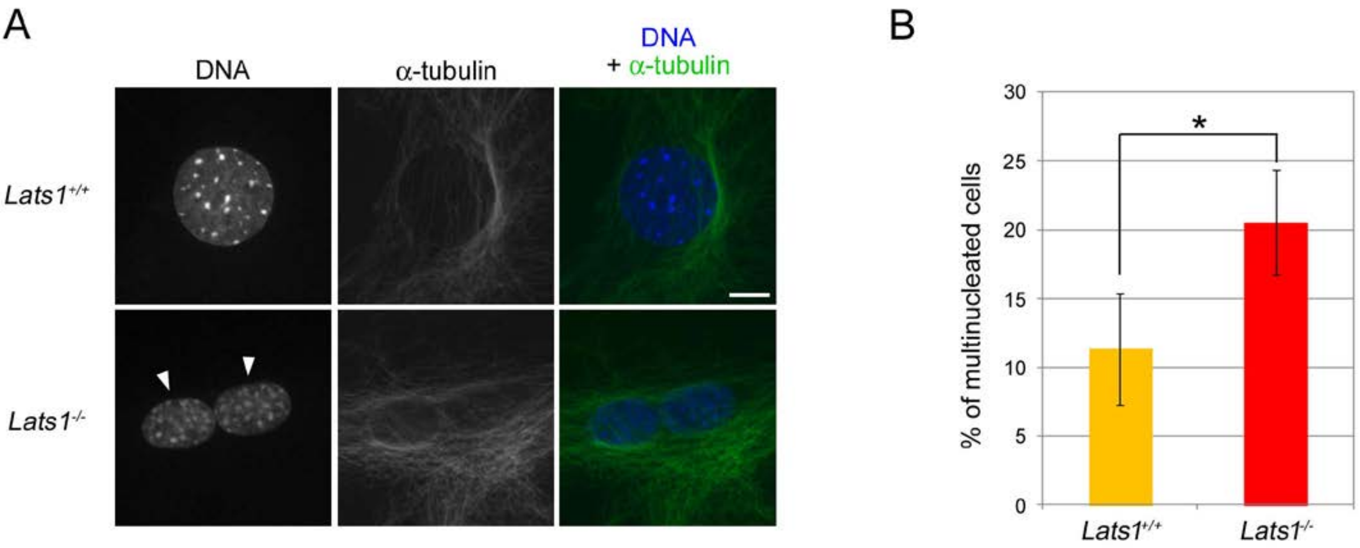

Figure S3. ***$Lats1^{-/-}$*** **MEFs exhibit a multinucleated phenotype.**

(A) *$Lats1^{+/+}$* and *$Lats1^{-/-}$* MEFs were stained with anti-α-tubulin antibodies (white or green) and Hoechst 33258 to detect DNA (white or blue). Arrowheads show binuclei in a cell. (B) The percentage of binuclear and multinuclear cells was assessed. Data represent the mean and standard deviation of three independent experiments. In each experiment, over 100 cells were counted.

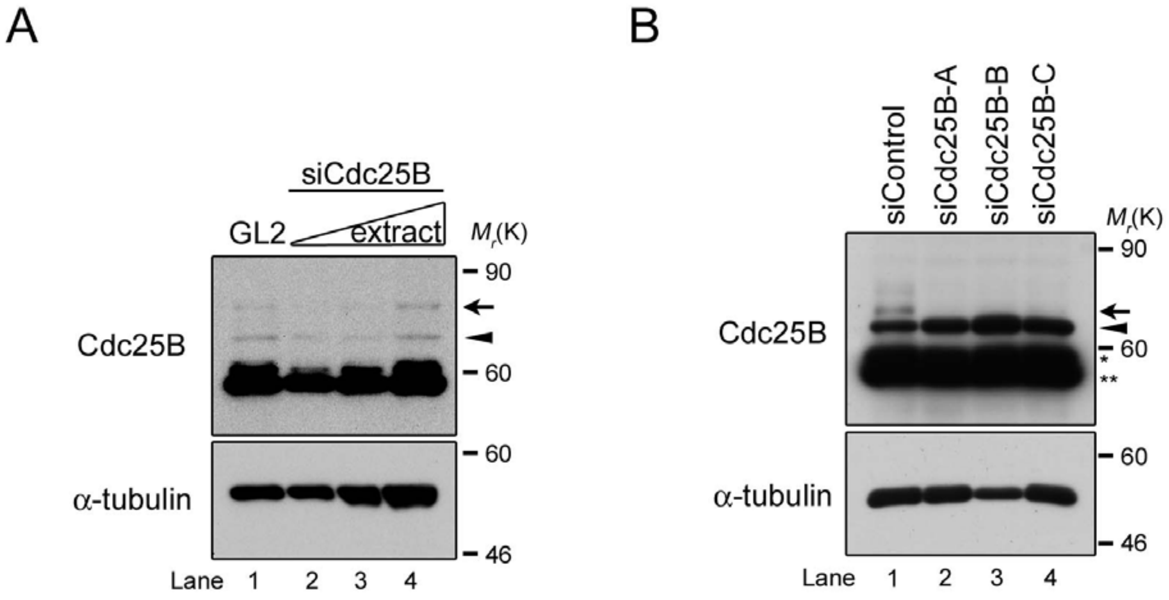

Figure S4. Cdc25B in *Lats1*$^{+/+}$ MEFs were reflected by upper band of two minor bands.

(A) *Lats1*$^{+/+}$ MEFs were transfected with siRNA duplex targeting Cdc25B or GL2 (negative control). Western blot analysis using serial dilution of the extract from Cdc25B knockdown. Proteins were detected using anti-Cdc25B and α-tubulin (loading control) antibodies. Arrow shows Cdc25B-specific bands. (B) *Lats1*$^{+/+}$ MEFs were transfected with three kinds of siRNA duplexes targeting Cdc25B (siCdc25B-A, -B, and -C). siControl was used as a negative control. Cdc25B was detected by western blotting as in A. Asterisks denote nonspecific bands. Refer to the text for detailed information about the bands indicated by the arrow and arrowhead.
